# Psychostimulants Modafinil, Atomoxetine and Guanfacine Impair Bone Cell Differentiation and MSC Migration

**DOI:** 10.3390/ijms231810257

**Published:** 2022-09-06

**Authors:** Nele Wagener, Wolfgang Lehmann, Lukas Weiser, Katharina Jäckle, Pietro Di Fazio, Arndt F. Schilling, Kai O. Böker

**Affiliations:** 1 Department of Trauma Surgery, Orthopedics and Plastic Surgery, University Medical Center Goettingen, Robert-Koch-Str. 40, 37099 Göttingen, Germany; 2 Department of Visceral Thoracic and Vascular Surgery, Philipps University Marburg, Baldingerstraße, 35043 Marburg, Germany

**Keywords:** bone defect, ADHD, hMSCs, cell migration, osteogenic differentiation, apoptosis

## Abstract

Attention deficit hyperactivity disorder (ADHD) is one of the most common worldwide mental disorders in children, young and adults. If left untreated, the disorder can continue into adulthood. The abuse of ADHD-related drugs to improve mental performance for studying, working and everyday life is also rising. The potentially high number of subjects with controlled or uncontrolled use of such substances increases the impact of possible side effects. It has been shown before that the early ADHD drug methylphenidate influences bone metabolism negatively. This study focused on the influence of three more recent cognitive enhancers, modafinil, atomoxetine and guanfacine, on the differentiation of mesenchymal stem cells to osteoblasts and on their cell functions, including migration. Human mesenchymal stem cells (hMSCs) were incubated with a therapeutic plasma dosage of modafinil, atomoxetine and guanfacine. Gene expression analyses revealed a high beta-2 adrenoreceptor expression in hMSC, suggesting it as a possible pathway to stimulate action. In bone formation assays, all three cognitive enhancers caused a significant decrease in the mineralized matrix and an early slight reduction of cell viability without triggering apoptosis or necrosis. While there was no effect of the three substances on early differentiation, they showed differing effects on the expression of *osterix* (*OSX*), *receptor activator of NF-κB ligand* (*RANKL*) and *osteoprotegerin* (*OPG*) in the later stages of osteoblast development, suggesting alternative modes of action. All three substances significantly inhibited hMSC migration. This effect could be rescued by a selective beta-blocker (Imperial Chemical Industries ICI-118,551) in modafinil and atomoxetine, suggesting mediation via beta-2 receptor stimulation. In conclusion, modafinil, atomoxetine and guanfacine negatively influence hMSC differentiation to bone-forming osteoblasts and cell migration through different intracellular pathways.

## 1. Introduction

More than 6 million children (9.4%) have been diagnosed with attention deficit hyperactivity disorder (ADHD) in the United States of America. The global prevalence of adult ADHD has been reported at 2.8% [1]. ADHD in children is characterized by the main symptoms of impaired concentration, hyperactivity and impulsivity and is one of the most common mental illnesses in children and adolescents [2]. The children affected by ADHD behave in a conspicuously uncontrolled, restless manner; show rash, ill-considered actions; do not follow instructions and rules; and show inappropriate reactions such as shouting and violence [2]. Recently, ADHD also has been introduced as an adult disorder. In 60% of children diagnosed with ADHD, the disorder persists into adulthood [3]. ADHD is commonly treated with the “cognitive enhancer” methylphenidate [4], as well as other drugs, including modafinil, atomoxetine and guanfacine, which are considered to increase concentration and alertness for learning and working [5]. Atomoxetine and guanfacine have been clinically approved for ADHD treatment [5,6]. The indications for modafinil include narcolepsy, obstructive sleep apnoea syndrome, chronic shift worker syndrome and sleep-associated disorders [7,8,9]. Off-label modafinil is also used for ADHD, Parkinson’s disease, chronic fatigue syndrome, jet lag, cognitive impairment in schizophrenia, eating disorders, depression, multiple sclerosis and cancer-related fatigue [7,8,9,10]. The current model of the mechanism of action of the three substances is characterized by an increase in noradrenaline and dopamine in the synaptic cleft in the central nervous system (CNS). The psychostimulant modafinil is thought to act as a central, selective alpha-1 agonist that causes an increase in the neurotransmitters dopamine and norepinephrine through presynaptic inhibition of the dopamine (DAT) and norepinephrine transporter (NET) [9,10]. Nevertheless, it is postulated that the arousal and activity-promoting effects of modafinil are largely a function in the catecholamine system with alpha and beta adrenergic receptor implication, but further studies are needed to understand the complicated mode of action of modafinil [9,10]. Guanfacine acts as a selective postsynaptic alpha-2 agonist and modulates synaptic norepinephrine transmission in the CNS [6]. Atomoxetine, a norepinephrine re-uptake inhibitor, increases norepinephrine levels by highly selectively inhibiting presynaptic NETs in the CNS [11]. 

The sympathetic nervous system controls heart rate, dilation of pupils or secretion of sweat glands but is also an important regulator in the homeostasis of bone formation and degradation. It promotes bone resorption and inhibits bone formation via beta-2 receptor activation on osteoblasts and osteocytes by noradrenaline. Osteoclast activation is mainly promoted indirectly via RANKL increase by osteoblasts [12,13,14,15,16].

The increasing number of prescriptions for ADHD medication indicates that more and more people are using the substances to mediate their mental performance [17,18]. It has already been shown that methylphenidate causes a growth reduction of 1.38 cm/year as well as a significant reduction in bone mineral density in 8–20 year olds [4], suggesting a similar effect of other drugs that act in a similar way on bone metabolism. Thus, this study investigates the molecular mechanisms of action of modafinil, atomoxetine and guanfacine regarding bone cell migration, osteogenic differentiation, cell viability and apoptosis/necrosis.

## 2. Results

### 2.1. Human Beta-2 Adrenoreceptor Is Most Highly Expressed Adrenoceptor in hMSCs and Human Osteoblasts (hOBs)

**Figure 1:**
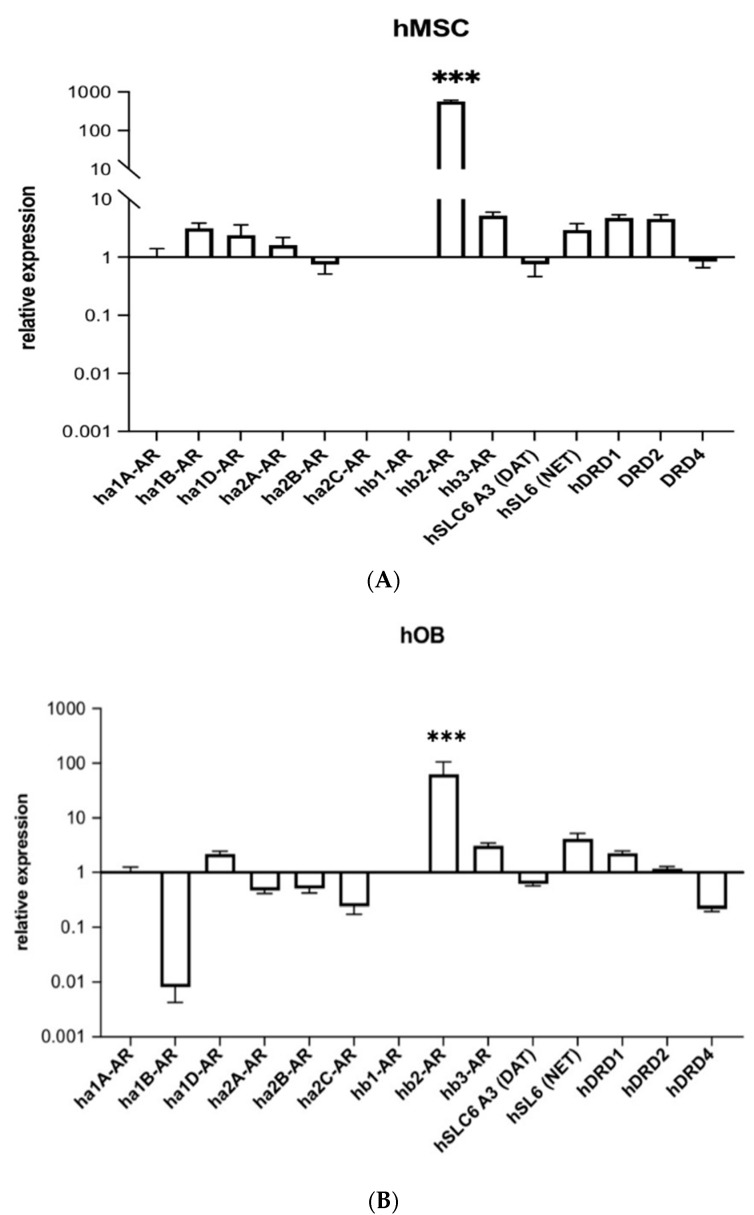
Transcript expression of human alpha1A-, alpha1B-, alpha1D-, alpha2A-, alpha2B-, alpha2C, beta1-, beta2-, beta3-, dopamine D1, D2, D4-receptors and dopamine- and noradrenaline transporters in hMSC (**A**) and hOB (**B**). The gene expressions were each normalized to ha1A-AR (relative gene expression) of hMSCs/hOBs. Shown are means ± standard deviation of three independent experiments with six biological replicates. Significance in difference between alpha-, beta-, dopamine receptors and dopamine- and noradrenaline transporter were determined by ANOVA followed by Tukey’s post hoc tests. *** corresponds to a value of *p* < 0.001.

HMSCs and hOBs were examined for expression of human alpha-1A/B/D, alpha-2A/B/C, beta-1/2/3, dopamine transporter (DAT), noradrenaline transporter (NET) and dopamine D1/2/4 receptors (Figure 1A,B). Both cell types showed the highest transcript level for the human beta-2 adrenoreceptor compared to other DAT and NET receptors.

### 2.2. ADHD-Medication Inhibits In Vitro Bone Formation

**Figure 2:**
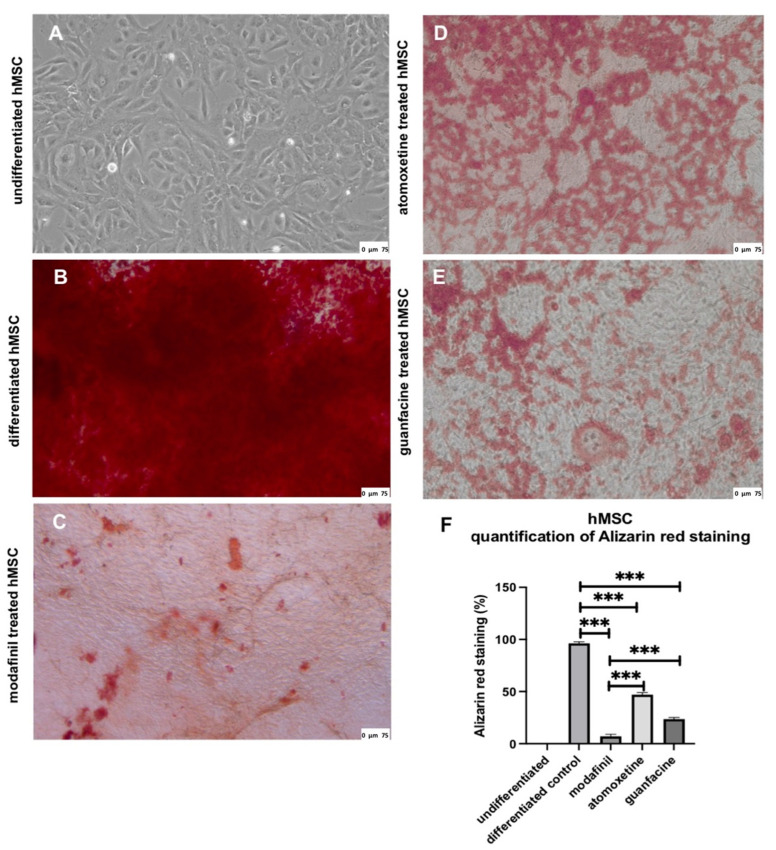
Alizarin red staining of undifferentiated hMSC (**A**) differentiated hMSC (**B**), modafinil (11.2 µg/mL) treated hMSC (**C**), atomoxetine (0.9 µg/mL) treated hMSC (**D**) and guanfacine (17.7 ng/mL) treated hMSC (**E**) for 28 days in 24-well plates of three independent experiments with six biological replicates. Osteogenic differentiation of hMSC significantly enhanced Alizarin red staining. Modafinil-, atomoxetine- and guanfacine-treated hMSC show a highly significant lower alizarin red staining after 28 days of osteogenic induction compared to differentiated hMSC. Densitometric quantification of Alizarin red staining in hMSC calculated by ImageJ software (**F**). Significance in difference between undifferentiated, differentiated, modafinil-, atomoxetine- and guanfacine treated hMSC was determined by ANOVA followed by Tukey’s post hoc tests. Scale bar 75 µm. *** corresponds to a value of *p* < 0.001.

Quantification of Alizarin red staining of extracellular calcium deposition showed, after 28 days of osteogenic induction, a strong presence of mineralized nodules in differentiated hMSCs (Figure 2B) compared to nondifferentiated cells (Figure 2A). Compared to the differentiated group without stimulant treatment, the extracellular matrix of hMSCs incubated for 28 days with modafinil (Figure 2C), atomoxetine (Figure 2D) and guanfacine (Figure 2E) displayed a highly significant decreased amount of red calcium nodules under light microscopic evaluation and quantification. Modafinil-treated hMSCs showed significantly lower alizarin red staining after 28 days compared to atomoxetine- and guanfacine-treated hMSCs (Figure 2F).

### 2.3. Modafinil, Atomoxetine and Guanfacine Do Not Cause Early Apoptosis and Secondary Necrosis in hMSCs

**Figure 3:**
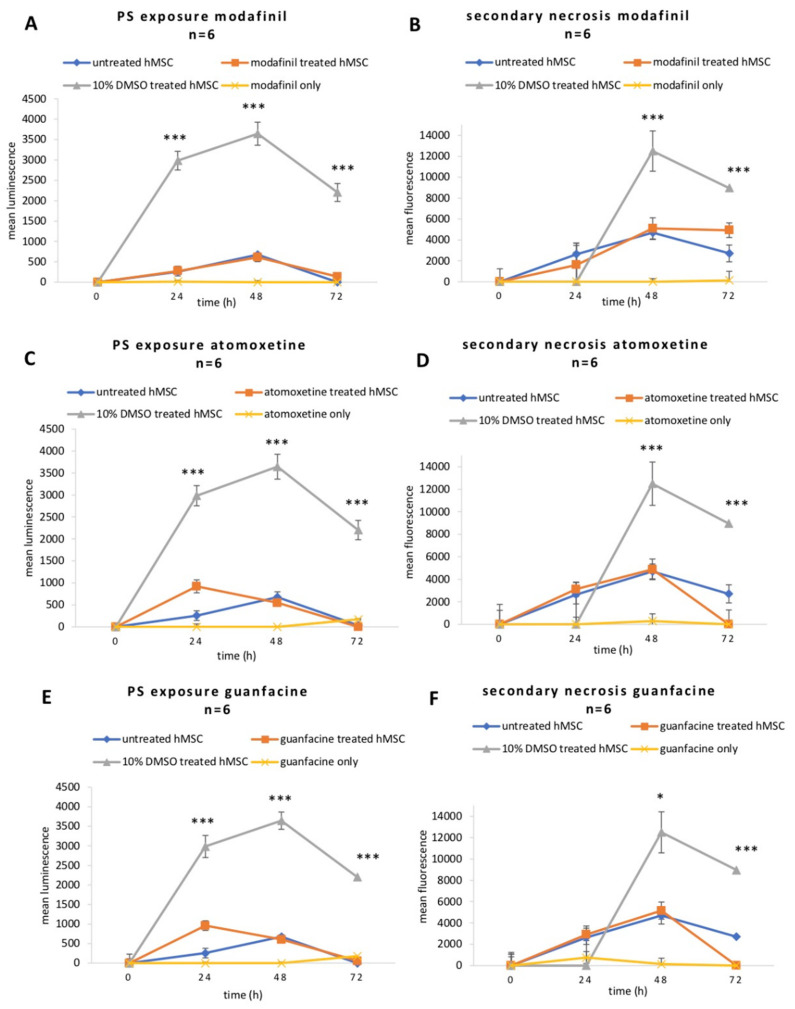
Detection of apoptosis/necrosis in hMSC after administration of modafinil (11.2 µg/mL) (**A**,**B**), atomoxetine (0.9 µg/mL) (**C**,**D**) and guanfacine (17.7 ng/mL) (**E**,**F**) for 24 h, 48 h and 72 h. Briefly, 10% DMSO was added to hMSC as a positive indicator of apoptosis/necrosis. The line diagram represents the luminescent signal of phosphatidylserine (PS) exposure and the fluorescent signal of secondary necrosis in hMSCs. Values are given as mean ± standard deviation to blank of three independent experiments with six biological replicates. Significance in difference between untreated, 10% DMSO-, modafinil-, atomoxetine- and guanfacine treated hMSC was determined by ANOVA followed by Tukey’s post hoc tests. * corresponds to a value of *p <* 0.05. *** corresponds to a value of *p <* 0.001.

There was no difference in apoptosis or secondary necrosis of hMSCs between the untreated and cognitive-enhancer-treated groups (Figure 3). Instead, the administration of 10% of DMSO caused an increase in the luminescence (phosphatidylserine exposure) and fluorescence (necrosis/DNA fragmentation), serving as a positive control for induced apoptosis in hMSCs.

### 2.4. Modafinil, Atomoxetine and Guanfacine Lead to Transient Decrease in Cell Viability

**Figure 4:**
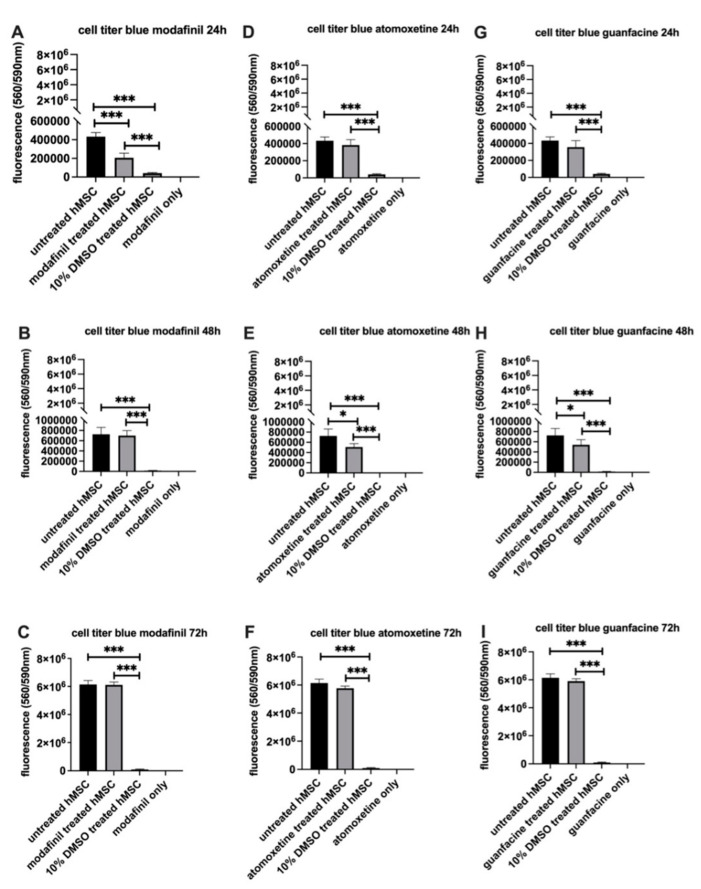
Quantification of cell viability of hMSC after administration of modafinil (11.2 µg/mL) for 24 h (**A**), 48 h (**B**), 72 h (**C**), atomoxetine (0.9 µg/mL) for 24 h (**D**), 48 h (**E**), 72 h (**F**) and guanfacine (17.7 ng/mL) for 24 h (**G**), 48 h (**H**), 72 h (**I**). Briefly, 10% DMSO was added to hMSC as a positive indicator of cell toxicity. Column height represents the metabolic capacity of hMSC to reduce resazurin into resorufin, which is fluorescent. Values are given as mean ± standard deviation to blank of three independent experiments with six biological replicates. Significance in difference between untreated, 10% DMSO-, modafinil-, atomoxetine- and guanfacine treated hMSC was determined by ANOVA followed by Tukey’s post hoc tests. * corresponds to a value of *p <* 0.05. *** corresponds to a value of *p <* 0.001.

Figure 4A–C shows that modafinil exhibits a highly significant reduction of cell viability compared to untreated hMSCs after 24 h of incubation. After 72 h of incubation with modafinil, cell viability was recovered to the values of the untreated control. The administration of atomoxetine and guanfacine (Figure 4D–I) did not cause a significant reduction of cell viability after 24 h, whereas after 48 h of incubation, a significant (*p* < 0.05) decrease in cell viability was caused by atomoxetine as well as guanfacine. After 72 h of incubation with atomoxetine and guanfacine, no reduction of cell viability in comparison to the untreated control was observed. As a positive control, 10% of DMSO caused a highly significant reduction of cell viability compared to untreated hMSCs at all time points.

### 2.5. Modafinil and Guanfacine Increase the Expression of RANKL and OSX after 28 Days

**Figure 5:**
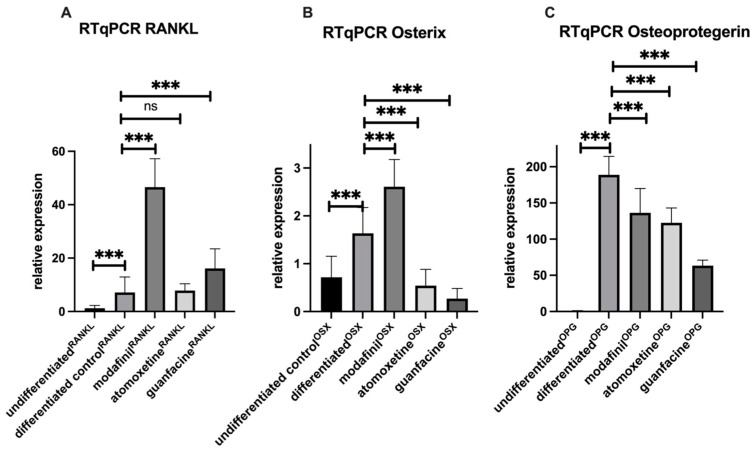
Expression of RANKL (**A**), OSX (**B**) and OPG (**C**) transcripts was determined in undifferentiated and osteogenic differentiated hMSC after the administration of modafinil (11.2 µg/mL), atomoxetine (0.9 µg/mL) and guanfacine (17.7 ng/mL) for 28 days by real-time quantitative PCR. Shown are means ± standard deviation of three independent experiments with six biological replicates. Significance in difference between the groups was determined by ANOVA followed by Tukey’s post hoc tests. *** corresponds to a value of *p <* 0.001. ns: not significant.

After 28 days of osteogenic stimulation, osteogenic differentiated hMSCs showed significant upregulation of expression of *RANKL*, *OSX* and *OPG* (* *p* < 0.001) compared to undifferentiated hMSCs (* *p* < 0.001) (Figure 5A–C). The administration of modafinil caused a highly significant further up-regulation of *RANKL* and *OSX*, as well as a highly significant down-regulation of *OPG* transcripts (Figure 5A–C). Atomoxetine caused no significant alteration of *RANKL* expression in comparison with the differentiated control group. However, a highly significant down-regulation of *OSX* and *OPG* transcript was detected. Guanfacine induced a highly significant upregulation of *RANKL* and downregulation of *OSX* and *OPG* transcripts in response to the differentiated control.

### 2.6. Osteogenic, Adipogenic and Chondrogenic Transcripts Are Not Affected by Psychostimulants after 7 Days of Osteogenic Differentiation

**Figure 6:**
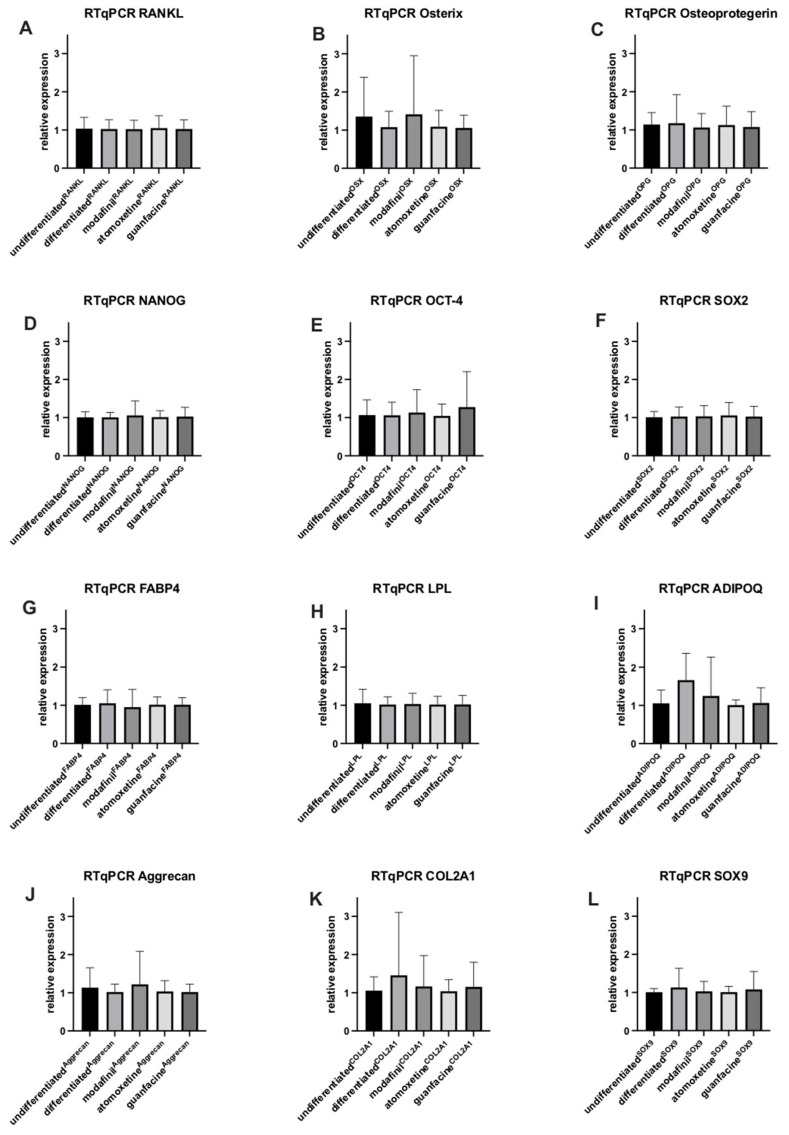
Expression of RANKL (**A**), OSX (**B**), OPG (**C**), NANOG (**D**), OCT-4 (**E**), SOX2 (**F**), FABP4 (**G**), LPL (**H**), ADIPOQ (**I**), Aggrecan (**J**), COL2A1 (**K**) and SOX9 (**L**) transcripts were determined in undifferentiated and osteogenic differentiated hMSC after the administration of modafinil (11.2 µg/mL), atomoxetine (0.9 µg/mL) and guanfacine (17.7 ng/mL) for 7 days by real-time quantitative PCR. Shown are means ± standard deviation of three independent experiments with six biological replicates. Significance in difference between the groups was determined by ANOVA followed by Tukey’s post hoc tests.

After 7 days of osteogenic stimulation, osteogenic differentiated hMSCs showed no significant up- and downregulation of expression of *RANKL*, *OSX*, *OPG NANOG*, *OCT-4*, *SOX2*, *FABP4*, *LPL*, *ADIPOQ*, *Aggrecan*, *COL2A1* and *SOX9* compared to undifferentiated hMSCs (Figure 6A–L). The administration of modafinil, atomoxetine and guanfacine caused no significant up- and downregulation of osteogenic, adipogenic, chondrogenic and stem cell differentiation transcripts (Figure 6A–L).

### 2.7. Modafinil, Atomoxetine and Guanfacine Inhibit hMSC Cell Migration

**Figure 7:**
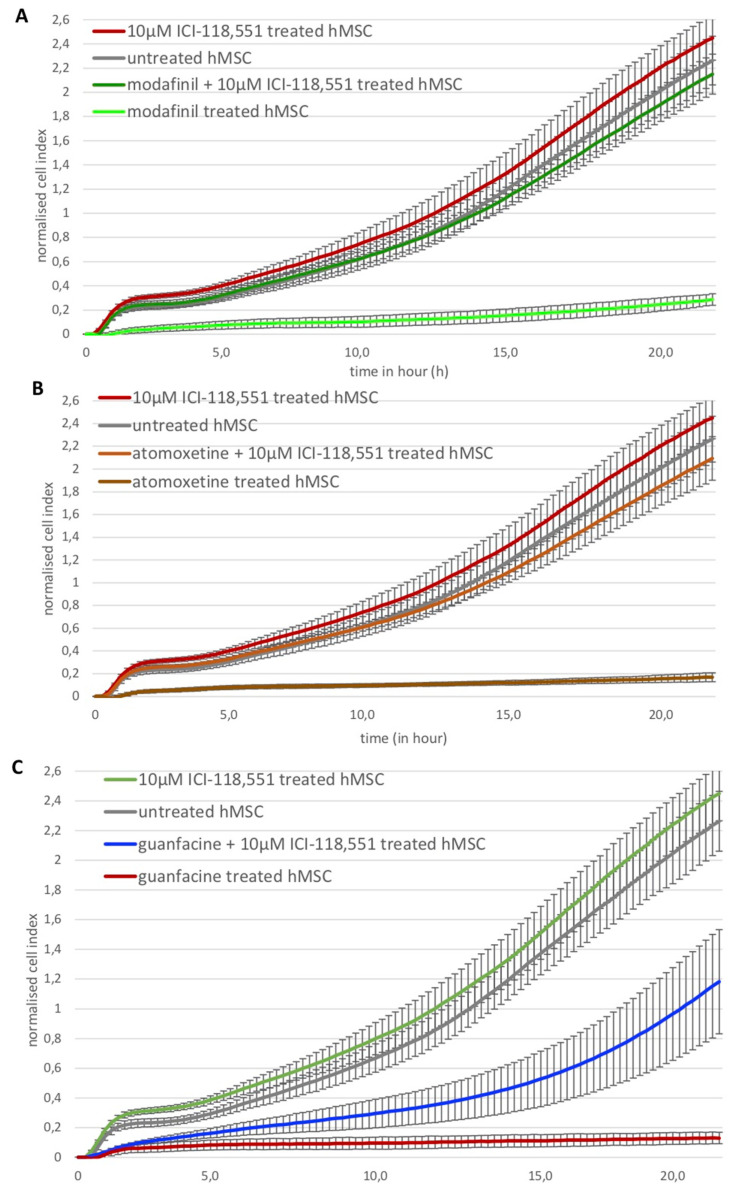
XCELLigence analysis of real-time cell migration of untreated hMSC and after the administration of modafinil (11.2 µg/mL), atomoxetine (0.9 µg/mL) and guanfacine (17.7 ng/mL) to hMSC for 24 h. XCELLigence measurement of real-time cell migration was performed after the administration of 10 µM ICI-118,551 to modafinil (11.2 µg/mL)-, atomoxetine (0.9 µg/mL)- and guanfacine (17.7 ng/mL) treated hMSC (**A**–**C**). Shown are means of normalized cell index ± SD of three independent experiments with six biological replicates.

Modafinil, atomoxetine and guanfacine were responsible for a significant reduction in cell migration of hMSCs (Figure 7A–C). This effect could be rescued by blocking beta2-adrenoreceptor through selective blocker ICI-118,551 at a concentration of 10 µM for modafinil and atomoxetine (Figure 7A,B), while the effect of guanfacine was not influenced by this (Figure 7C). ICI-118,551 alone did not show any significant effect at the used concentration (Figure 7A–C).

## 3. Discussion

Currently, no in vitro research is available on the impact of the three psychostimulants modafinil, atomoxetine and guanfacine on cell viability, apoptosis/necrosis, cell migration and the osteogenic differentiation of hMSCs. 

The current state of research on modafinil, atomoxetine and guanfacine is limited to Pal China et al., who reported an osteocatabolic impact of modafinil in Sprague-Dawley rats [19].

The study aimed to analyze the effect of three cognitive enhancers, modafinil, atomoxetine and guanfacine, on bone tissue. It has been shown before that methylphenidate, another cognitive enhancer inhibiting the DAT and NET system, causes a growth reduction of 1.38 cm/year and decreases the mineral density of bone tissue [4]. This effect was attributed to increased osteoclast activity, as seen in a rat model [4]. Up to now, the effect of modafinil, atomoxetine and guanfacine on bone tissue is unclear. To evaluate the ability of modafinil, atomoxetine and guanfacine to pertub the physiology of bone cells, the expression of NET and DAT receptors in hMSCs was initially monitored. Transcript expression of human beta-2 adrenoreceptor was strongly increased compared to other receptors, in hMSC and in hOB. Furthermore, we could detect relevant amounts of the majority of alpha- and beta adrenoreceptors (ha1-AR, ha1B-AR, ha1D-AR, ha2-AR and hB3-AR) and also the norepinephrine transporter (NET) and the dopamine transporters D1 and D2. These results are consistent with the findings of Ma et al. [20], who found NET expression in human bone marrow MSCs and calvaria-isolated primary osteoblasts. Furthermore, Tyurin-Kuzmin et al. showed alpha1A, alpha1B, alpha2A, alpha2B, beta 1, beta 2 and beta 3 adrenergic receptor expression on the MSC surface [21].

Nevertheless, a decrease in ha1B-AR expression was observed in human osteoblasts compared to mesenchymal stem cells. These results support the findings of Huang and colleagues showing the expression of alpha1B-AR, alpha1D-AR, alpha2A- and beta2-AR in osteoblasts. Noradrenaline exerted in those cells a proliferative effect via alpha1-AR and an antiproliferative effect via beta2-AR [22].

The described receptor expression in bone cells allows us to hypothesize a possible action of modafinil, atomoxetine and guanfacine on both mesenchymal stem cells and osteoblasts. Therefore, we analyzed the influence of the three cognitive enhancers on osteogenic differentiation. We have deliberately focused on desmal osteogenesis in this work, which involves the differentiation of mesenchymal cells directly into osteoblasts, which are responsible for the production of bone tissue (ossification). Modafinil and guanfacine caused a significant upregulation of the transcript of *RANKL* and significant downregulation of the *OPG* transcript. 

*OSX* transcripts were significantly upregulated by modafinil, whereas atomoxetine and guanfacine caused a significant downregulation on 28th day of incubation. Osterix functions as an osteoblast-specific transcription factor activating specific genes during osteogenic differentiation of preosteoblasts to mature osteoblasts and was therefore analyzed in this study. For example, Limraksasin et al. showed the highest significant upregulation of Osterix on days 36 and 43 of osteogenic differentiation, whereas this was absent on days 7 and 14 of differentiation [23].

Atomoxetine-treated hMSCs showed stable *RANKL* expression and highly significant OPG downregulation. Inside bone, these combined phenotypes would result in increased differentiation of osteoclasts with subsequent bone resorption. For methylphenidate, similar results were reported in a rat model, where this cognitive enhancer leads to increased osteoclast differentiation, activity and resorption [4]. Interestingly, this study evidenced that the osteogenic differentiation of hMSCs and the mineralization were effectively impaired after treatment with modafinil, atomoxetine and guanfacine. 

Similarly, Pal China and colleagues showed that modafinil induced trabecular and cortical bone loss in 8-week-old Sprague Dawley rats [19]. Ben-Ami and colleagues were able to show in a retrospective cohort study of 689 soldiers with diagnosed ADHD that methylphenidate is associated with an increased risk of stress fractures, whereas traumatic fractures are reduced [24]. Stress fractures can occur when enhanced osteoclastic resorption overwhelms osteoblastic formation activity [25], which is in line with the above-described *RANKL* increase and *OPG* decrease, thus leading to osteoclast activation. Schermann and colleagues confirmed the research findings of Ben-Ami et al. showing that the reduction of bone mineral density (BMD) induced by methylphenidate was associated with an increased incidence of stress fractures in soldiers based on a case-control study [26]. Chen and colleagues also showed that ADHD patients treated with methylphenidate have a lower risk of traumatic fractures [27]. This might possibly be explained by the fact that the assumption of a high dosage of psychostimulants might avoid casual accidents. 

To shed some light on the mechanism of bone formation inhibition, we compared the effects of the cognitive enhancers on cell viability and apoptosis/necrosis, cell migration and the osteogenic differentiation of differentiated bone cells derived from hMSCs. Despite a significant reduction of cell viability observed after the administration of the maximum therapeutic plasma concentration of modafinil, atomoxetine and guanfacine, neither induction of apoptosis nor necrosis could be observed in hMSCs. Cell viability recovered within 72 h after treatment with modafinil, atomoxetine and guanfacine. In comparison, methylphenidate, a piperidine-derived drug for ADHD patients, causes cytotoxic effects already at a concentration of 90 µM [28], which is below the maximum therapeutically used dose (60 mg methylphenidate) of 103 µM in the brain [29]. Kaya and colleagues evidenced that the administration of methylphenidate to primary nucleus pulposus cells (NPCs) and annulus fibrosus cells (AFCs) led to a reduction in cell viability and cell proliferation [30]. Instead, this study evidenced that all the administered compounds highlighted a short time efficacy on the cell viability, which was completely recovered 72 h after the administration.

Analogous to our results, cytotoxicity of modafinil could be excluded by de Castro Penna and colleagues in 2017 [31] using a daily dose of 0.75 mg/mL in bone marrow cells from Wistar rats. Novotna and colleagues in 2014 also ruled out cytotoxicity at concentrations above 100 µM of modafinil on AZ-AhR cells derived from human HepG2 liver cells [32]. Lee et al. showed a short-term decrease in cell viability after doxorubicin treatment in human gingiva derived stem cells leading to a reduced osteogenic differentiation capacity [33], which is comparable to our observations of short time inhibition of cell viability of all compounds, followed by decreased osteogenic differentiation. Since we observed a significantly decreased amount of red calcium nodules for all cognitive enhancers after 28 days of osteogenic differentiation, we decided to test if there is an alternative differentiation route forced by the medication or if cells stay in a more stem-cell-like state. After 7 days of osteogenic differentiation, no cognitive enhancer changed the expression of adipogenic and chondrogenic marker proteins, indicating no alternative differentiation route through the medication. Furthermore, osteogenic differentiation was unaffected, suggesting a late-stage effect of the cognitive enhancers on this differentiation route. These results were supported by the analysis of stem cell markers NANOG, OCT-4 and SOX2. All markers stay unaffected after 7 days of differentiation, which demonstrates the stem-cell-like state is still present after cognitive enhancer medication for 7 days in an osteogenic environment.

Next, the effect of ADHD medication was monitored on cell migration. Interestingly, modafinil, atomoxetine and guanfacine abolished the cell migration. As stem cell migration has been shown to be important in bone formation [34], wound healing [35] and bone remodeling/repair [36], it can be assumed that administration of the compounds may influence these processes, therefore possibly also affecting healthy people abusing the above substances on a daily basis for cognitive enhancement. On the other hand, studies have shown that untreated children with ADHD have a significantly increased risk of fracture compared to children without ADHD (hazard ratio 1.64, 95% confidence interval). Ritalin treatment decreased the risk in this population to normal levels (hazard ratio 1.12, 95% confidence interval), suggesting a net-positive influence of Ritalin on bone health in ADHD children [37]. Nevertheless, the influence of Ritalin on healthy people (abuse) remains unclear. It has been shown before that beta-2 adrenoreceptor activation modulated cell migration, for example, in dermal fibroblasts that are critical for wound repair [38]. Wnorowski and colleagues were able to show that the cell migration and the proliferation of human melanoma cells were inhibited by the beta-2 adrenoceptor agonist *R*,R′-4′-methoxy-1-naphthyl fenoterol [(*R*,*R*′)-MNF]. The inhibition of their migration could also be reversed by the administration of the highly selective beta-2-adrenoceptor antagonist ICI-118,551 (50 nM) [39].

In contrast, Iseri and colleagues showed that the highly selective beta-2 antagonist ICI-118,551 inhibits cell migration, invasion and proliferation of MCF7, HT-29 and HepG2 depending on concentration and exposure time [40].

Bravo Calderon and colleagues showed that beta-adrenergic receptor activation of two cancer cell lines inhibits cancer cell invasion and migration [41].

The evidence of the expression of the gene encoding for the human beta-2 adrenoreceptor, highlighted in hMSCs and hOBs, gives a possible pathway for the ability of modafinil to reduce cell migration within 24 h of treatment. Indeed, hMSCs were able to migrate again in the presence of modafinil or atomoxetine after the administration of the highly selective receptor blocker of the human beta-2 receptor. Our results suggest that modafinil and atomoxetine do not only have central effects on the CNS but direct peripheral effects on bone cells through a distinct beta-2 activity, additionally to the already described alpha-1 activity of modafinil [7] and other mechanisms [9]. The pathway of inhibition of migration by guanfacine remains unresolved since it was not affected by the beta-2 receptor antagonist ICI-118,551. Our results confirm that the sympathetic nervous system [12] and the neurotransmitters norepinephrine and epinephrine could play a significant role in bone defect regeneration, as hMSCs as well as hOBs indeed express relevant amounts of the majority of alpha- and beta adrenoceptors (ha1-AR, ha1B-AR, ha1D-AR, ha2A-AR and hB3-AR) and also the norepinephrine transporter (NET) and the dopamine transporters D1 and D2.

The current study is limited by the fact that the human metabolism of the ADHD drug used is not considered due to the in vitro study design. It is further limited by the short-term nature of the experiments. In future studies, it would be interesting to further investigate the effects of recent ADHD medication on osteoblast differentiation (e.g., bALP staining or Col1 production) but also study the effect on osteoclast differentiation. Furthermore, the effects of these cognitive enhancers on chondrogenic differentiation should be studied to obtain more insights into the influence on bone healing and growth retardation in young adults.

Future studies in vivo would be desirable, possibly using a fracture healing model in the mouse/rat to simulate the homing function of the hMSCs and studying the influence on bone healing via in-vivo CT analysis and bone histology of different stages of bone healing. It still remains unclear whether modafinil increases *RANKL* and *OSX* but decreases *OPG* and inhibits bone formation in general. Furthermore, the different modes of action of guanfacine (not affected by beta-2 blocker) should be analyzed in more detail.

## 4. Materials and Methods

### 4.1. Cell Culture

Single-cell-derived human mesenchymal stem cell lines expressing hTERT (SCP1 cell line) [42] were maintained in Dulbecco’s Modified Eagle’s Medium (DMEM) low glucose medium with 10% fetal calf serum (FCS) and 1% antibiotics penicillin and streptomycin, (PS). Human osteoblastic-like MG63 cells were obtained from CLS (Cell Line Service, Eppelheim, Germany, product no. 300441) and cultured in DMEM-F12 (Gibco, Paisley, UK), with the addition of 10% FCS and 1% PS. (Gibco, Paisley, UK). All cells were incubated according to standard conditions in a humidified atmosphere at 37 °C and 5% CO_2_.

### 4.2. Substances

Modafinil solution (1 mg/mL), atomoxetine hydrochloride solution (1 mg/mL) and guanfacine hydrochloride (500 mg/mL) were dissolved in sterile dimethyl sulfoxide (DMSO). ICI-118,551-hydrochloride powder was dissolved in sterile water (10 mg/mL). All substances were purchased from Sigma-Aldrich (Steinheim, Germany). The concentrations of modafinil (11.2 µg/mL), atomoxetine (0.9 µg/mL) and guanfacine (17.7 ng/mL) were based on the maximum therapeutic plasma concentrations [11,43,44], and 10% DMSO was obtained from Merck, Germany. Ascorbic acid-2 phosphate (200 µM, Cayman Chemical, Ann Arbor, MI, USA), dexamethasone (0.1 µM) and ß-glycerol phosphate (10 mM, Merck, Darmstadt, Germany) were applied for osteogenic differentiation.

### 4.3. Osteogenic Differentiation

Osteogenic induction of hMSCs was performed for 7 and 28 days with osteogenic differentiation medium (ODM, DMEM low-glucose containing 10% FCS, 1% Pen/Strep., 200 µM ascorbic acid-2-phosphate, 0.1 µM dexamethasone and 10 mM ß-glycerolphosphate) (Table 1).

### 4.4. RNA Isolation and Quantitative RT-PCR

HMSCs (50.000/well) were seeded in 24-well plates with the addition of osteogenic differentiation medium (ODM) (Table 1), modafinil (11.2 µg/mL), atomoxetine (0.9 µg/mL) and guanfacine (17.7 ng/mL) for an incubation period of 28 days. Ribonucleic acid (RNA) extraction was performed using Trizol reagent (Invitrogen, Waltham, MA, USA) according to the manufacturer’s instructions. RNA quality and concentration were measured using DS-11 FX with an integrated spectrophotometer (Thermo Fisher Scientific, Waltham, MA, USA). For quantitative analysis of mRNA, 1000 ng of RNA was reverse transcribed and amplified using the iScript^TM^ cDNA Synthesis Kit (170-8891, Bio-Rad Laboratories, Hercules, CA, USA) using a thermocycler (Labcycler, SensoQuest, Göttingen, Germany). Primer sequences for adrenoreceptors [45], DAT [46] and dopamine receptors [45] were checked in the literature, while sequences for OPG (148743792c1), RANKL (197927084c1), SP7 (22902135c1), NANOG, OCT-4, SOX2, FABP4, LPL, ADIPOQ, Aggrecan, COL2A1 and SOX9 were described in the openly accessible primerbank [47,48,49]. Oligonucleotides were synthesized by Microsynth AG (Balgach, Switzerland). Primer sequences are listed in Table 2 and Table 3. 

Quantitative RT-PCR was performed using the SsoAdvanced Universal SYBR Green Super mix System (Bio-Rad Laboratories, Hercules, CA, USA) and the RT-qPCR thermocycler CFX96^TM^ Real-Time System (Bio-Rad Laboratories, CA, USA). Results were analyzed with the Bio-Rad CFX Manager (Bio-Rad Laboratories) and normalized with mRNA GAPDH content for each sample. All calculations for the relative results were conducted using the standard 2^−∆∆CT^ method using ha1A-AR as a reference (expression equal to 1).

### 4.5. Cell Titer-Blue Cell Viability Assay

Cell viability measurement using Cell Titer Blue is performed by adding the redox dye resazurin, which is metabolized by viable cells into fluorescent resofurin. Non-viable cells show a suspended metabolic activity so that fluorescence signals are absent. After 48 h of cell seeding, modafinil, atomoxetine, guanfacine and 10% DMSO were added because, at this point, cellular confluence of 70% was approached in each of the 96-well plates. The Cell Titer blue was performed to determine the possible cytotoxicity of these compounds after a short time treatment in terms of reduction of cell viability. 

Quantification of cell viability was measured by fluorescence after 24 h, 48 h and 72 h administration of the maximum therapeutic plasma concentration of modafinil (11.2 µg/mL), atomoxetine (0.9 µg/mL) and guanfacine (17.7 ng/mL) by using CellTiter-Blue Reagent (G8080, Promega, Madison, WI, USA). 

HMSCs (10.000/well) were seeded in 96-well plates. After 48 h, modafinil, atomoxetine, guanfacine and 10% DMSO were added. The measurement was carried out using a plate reader (Victor, PerkinElmer, Waltham, MA, USA) after an incubation period of 24 h, 48 h and 72 h. Data were analyzed using Excel 2022 (Microsoft, Redmond, WA, USA).

### 4.6. Measurement of Apoptosis/Necrosis

Early apoptosis and secondary necrosis were detected by luminescence/fluorescence after the addition of the Real Time-Glo^TM^ Annexin V Apoptosis and Necrosis Assay (JA1011 Promega, Madison, WI, USA). hMSCs (10.000/well) were seeded into a 96-well plate. After 48 h, the substances modafinil (11.2 µg/mL), atomoxetine (0.9 µg/mL) and guanfacine (17.7 ng/mL) were added. The measurement was conducted using a plate reader (Victor, PerkinElmer, Waltham, MA, USA) after an incubation period of 24 h, 48 h and 72 h. Data were analyzed using Excel 2022 (Microsoft).

### 4.7. Real-Time Cell Migration Analysis

HMSCs (40,000/well) were seeded in Real-Time Cell Analysis (RTCA) CIM-Plate 16 (OLS, Bremen, Germany), and real-time cell migration was monitored for 24 h after the administration of the maximum therapeutic plasma concentration of modafinil (11.2 µg/mL), atomoxetine (0.9 µg/mL) and guanfacine (17.7 ng/mL) and the human beta-2 receptor antagonist ICI-118,551 by xCELLigence RTCA DP system (Roche, Basel, Switzerland).

In preliminary experiments, ICI-118,551 was tested at concentrations of 10, 50 and 100 µM, while a concentration of 10 µM was sufficient for this experiment. XCELLigence performs real-time impedance-based cell migration measurements using electronically integrated Boyden chambers. The migration of cells from the upper chamber was registered by impedance sensors of a micropore membrane and after adherence at the bottom of the lower chamber by impedance measurements of a microelectrode.

### 4.8. Histochemical Stains

First, seeding of hMSCs (50,000/well) was conducted in 24-well plates. After reaching a cell confluence of more than 70%, the administration of osteogenic differentiation medium (ODM) (Table 1), modafinil (11.2 µg/mL), atomoxetine (0.9 µg/mL) and guanfacine (17.7 ng/mL) was performed for an incubation period of 28 days. 

Alizarin red staining was used to detect calcium phosphate of osteogenic differentiated hMSCs. To prepare the staining solution, 2 g of alizarin red-S was dissolved in 100 mL of distilled water with constant stirring. Before using the staining solution, the pH was adjusted to 4.1–4.3 using 10% ammonium hydroxide, and then the solution was filtered.

Extracellular calcium deposition was detected by light microscopy (Leica DMi8, Wetzlar, Germany), and densitometric quantification was performed using ImageJ software.

### 4.9. Software and Statistical Analysis

Significance in difference between undifferentiated, differentiated, modafinil-, atomoxetine- and guanfacine treated hMSC was determined by ANOVA followed by Tukey’s post hoc tests. Data analysis was performed using Excel 2022 (Microsoft) and GraphPad Prism 9. All results show means, including standard deviation. Statistical significance is indicated with asterisks (* *p* < 0.05; ** *p* < 0.01; *** *p* < 0.001); * corresponds to a value of *p* < 0.05, ** corresponds to a value of *p* < 0.01, and *** corresponds to a *p*-value of *p* < 0.001. All experiments were repeated with three technical and six biological replicates.

## 5. Conclusions

Therapeutic plasma concentration of modafinil, atomoxetine and guanfacine impaired the cell migration of hMSCs, for modafinil and atomoxetine via beta-2 agonism. Modafinil, atomoxetine and guanfacine caused a significantly decreased matrix calcification without promotion of apoptotic cell death. Additionally, modafinil and guanfacine showed an indirect osteocatabolic effect by upregulating the molecules necessary for osteoclastogenesis.

## Figures and Tables

**Table 1:** Osteogenic differentiation medium.

Component	Volume/Concentration	Company
DMEM (low glucose)	500 mL	Sigma Aldrich, Taufkirchen, Germany
Fetal calf serum (FCS)	50 mL (10%)	PAN Biotech, Aidenbach, Germany
Penicillin/Streptomicin	5 mL (1%)	PAN Biotech, Aidenbach, Germany
Ascorbic acid-2 phosphate	200 µM	Cayman chemical company, Ann Arbor MI, USA
ß-glycerolphosphate	10 mM	Carl Roth, Karlsruhe, Germany
Dexamethasone	0.1 µM	Carl Roth, Karlsruhe, Germany

**Table 2:** Primer sequences for real-time qPCR assessment.

Gene	Primer Sequences
*Glyceraldehyde-3-phosphate dehydrogenase (GAPDH)*	For: 5′-AGGTCGGAGTCAACGGAT-3′
	Rev: 5′-TCCTGGAAGATGGTGATG-3′
*α_1_A-adrenoreceptor* (*ADRA1A)*	For: 5′-ATCATCTCCATCGACCGCTACA-3′
	Rev: 5′-TCACTTGCTCCGAGTCCGACTT-3′
*α* *_1_* *B-adrenoreceptor (ADRA1B)*	For: 5′-CCATTCCAAGAACTTTCACGA-3′
	Rev: 5′-CCAGAACACCACCTTGAACAC-3′
*α* *_1_* *D-adrenoreceptor (ADRA1D)*	For: 5′-TCTTTTCGGGGTGCTGGGTAA-3′
	Rev: 5′-TGGGTGACGATGGTTGGGTAG-3′
*α_2_A-adrenoreceptor* (*ADRA2A)*	For: 5′-TCGTCATCATCGCCGTGTTCA-3′
	Rev: 5′-GCCGCCGCCGCCCTTCTTCTC-3′
*α_2_B-adrenoreceptor* (*ADRA2B)*	For: 5′-GGGAGACCCCTGAAGATACTG-3′
	Rev: 3′-ACAAAAACGCCAATGACCACA-5′
*α_2_C-adrenoreceptor* (*ADR2C)*	For: 5′-GTGGTGATCGCCGTGCTGAC-3′
	Rev: 5′-CGTTTTCGGTAGTCGGGGAC-3′
*b_1_-adrenoreceptor (ADRB1)*	For: 5′-GCCATCGCCTCGTCCGTAGTC-3′
	Rev: 5′-CGTAGCCCAGCCAGTTGAAGA-3′
*b_2_-adrenoreceptor (ADRB2)*	For: 5′-TCTGATGGTGTGGATTGTGTC-3′
	Rev: 5′-ACGTCTTGAGGGCTTTGTGCT-3′
*b_3_-adrenoreceptor (ADRB3)*	For: 5′-CCCAATACCGCCAACACCAGT-3′
	Rev: 5′-CGACCCACACCAGGACCACAG-3′
*Dopamine transporter 1 (SLC6A3)*	For: 5′-CGGCCAGACCAAGAGGGAAGAAGCA 3′
	Rev: 5′-TGGGCACACTGGGAGTTGAGGAA-3′
*Norepinephrine transporter (SLC6A2)*	For: 5′-GGCGTTGGCTATGCTGTCAT-3′
	Rev: 5′-AGCTTGGGGTCGGTACAGTT-3′
*Dopamine receptor 1 (DRD1)*	For: 5′-TGGTCTGTGCTGCCGTTATCAG-3′
	Rev: 5′-CAATCTCAGCCACTGCCTTCCA-3′
*Dopamine receptor 2 (DRD2)*	For: 5′-CAATACGCGCTACAGCTCCAAG-3′
	Rev: 5′-GGCAATGATGCACTCGTTCTGG-3′
*Dopamine transporter 4 (DRD4)*	For: 5′-TGCTGCCGCTCTTCGTCTACTC-3′
	Rev: 5′-ACAGGTTGAAGATGGAGGCGGT-3′

**Table 3:** Primer sequences for real-time qPCR assessment.

Gene	Primer Sequences
*GAPDH*	For: 5′-AGGTCGGAGTCAACGGAT-3′
	Rev: 5′-TCCTGGAAGATGGTGATG-3′
*Osteoprotegerin (TNFRSF11B)*	For: 5′-GCGCTCGTGTTTCTGGACA-3′
	Rev: 5′-AGTATAGACACTCGTCACTGGTG-3′
*RANKL (TNFSF11)*	For: 5′-CAACATATCGTTGGATCACAGCA-3′
	Rev: 5′-GACAGACTCACTTTATGGGAACC-3′
*Osterix (SP7)*	For: 5′-CCTCTGCGGGACTCAACAAC-3′
	Rev: 5′-AGCCCATTAGTGCTTGTAAAGG -3′
*Nanog Homeobox* *(NANOG)*	For: 5′-ACCTCAGCCTCCAGCAGATG-3′
	Rev: 5′-TGCACCAGGTCTGAGTGTTC-3′
*Octamer binding transcription factor 4* *(OCT-4)*	For: 5′-GATCACCCTGGGTATACAC-3′
	Rev: 5′-GCTTTGCATATCTCCTGAAG-3′
*Sex-determining region Y (SRY)-box 2 (* *SOX2)*	For: 5′-ATGGGTTCGGTGGTGGTCAAG-3′
	Rev: 5′-GGCAGTGTGCCGTTAATG-3′
*Fatty acid-binding protein 4 (FABP4)*	For: 5′-ACTGGGCCAGGAATTTGACG-3′
	Rev: 5′-CTCGTGGAAGTGACGCCTT-3′
*Lipoprotein lipase (* *LPL)*	For: 5′-TCATTCCCGGAGTAGCAGAGT-3′
	Rev: 5′-GGCCACAAGTTTTGGCACC-3′
*Adiponectin, C1Q And Collagen Domain Containing* *(ADIPOQ)*	For: 5′-AACATGCCCATTCGCTTTACC-3′
	Rev: 5′-TAGGCAAAGTAGTACAGCCCA-3′
*ACAN (Aggrecan)*	For: 5′-GTGCCTATCAGGACAAGGTCT-3′
	Rev: 5′-GATGCCTTTCACCACGACTTC-3′
*Collagen Type II Alpha 1 Chain (* *COL2A1)*	For: 5′-CCAGATGACCTTCCTACGCC-3′
	Rev: 5′-TTCAGGGCAGTGTACGTGAAC-3′
*SRY-Box Transcription Factor* *9* *(SOX9)*	For: 5′-AGCGAACGCACATCAAGA-3′
	Rev: 5′-CTGTAGGCGATCTGTTGGGG-3′

## Data Availability

Not applicable.
